# Dentinal Tubule Penetrability and Bond Strength of Two Novel Calcium Silicate-Based Root Canal Sealers

**DOI:** 10.3390/ma16093309

**Published:** 2023-04-23

**Authors:** Karissa Shieh, Jack Yang, Elsa Heng Zhu, Ove Andreas Peters, Sepanta Hosseinpour

**Affiliations:** School of Dentistry, The University of Queensland, Brisbane 4006, Australia

**Keywords:** penetration, bond strength, confocal, push-out test, calcium silicate-based sealer

## Abstract

Background: Once the chemo-mechanical preparation of root canals is finished, achieving a complete seal of the root canal system becomes crucial in determining the long-term success of endodontic treatment. The important goals of root canal obturation are to minimize leakage and achieve an adequate seal. Thus, a material that possesses satisfactory mechanical characteristics, is biocompatible, and has the ability to penetrate the dentine tubules adequately is needed. Aim: This study aimed to compare the penetrability and bond strength between two calcium silicate-based sealers and an epoxy resin-based sealer, as well as examine the relationship between penetrability and bond strength for the different sealers. Method and materials: Thirty-nine recently extracted single-rooted human premolar teeth were instrumented and divided evenly into three groups (*n* = 13), according to the sealer used for obturation: AH Plus Jet, EndoSequence, and AH Plus Bioceramic Sealer. Three teeth (30 slices) were randomly selected out of each for analysis using confocal laser scanning microscopy to assess penetrability. The remaining ten teeth (90 slices) in each group were subject to push-out tests using a universal testing machine. All teeth were sectioned into nine transverse slices of 0.9 mm thickness for their respective tests (apical, middle, coronal). Results: AH Plus Jet exhibited significantly lower penetrability and significantly higher bond strength compared to EndoSequence BC sealer (*p* = 0.002) and AH Plus Bioceramic Sealer (*p* = 0.006). There was no significant difference between EndoSequence BC sealer and AH Plus Bioceramic Sealer in terms of either penetrability or bond strength. No correlation was found between penetrability and bond strength. Conclusions: Within the limitation of this study and regardless of the location in the canal, the bioceramic based root canal sealers appeared to perform better than the epoxy resin-based sealer in terms of dentinal penetration rate. Further studies are required to compare other biomechanical properties of bioceramic sealers including setting characteristics and bacterial leakage.

## 1. Introduction

Successful endodontic therapy is contingent on the proper disinfection and complete obturation of the root canal system [1]. Root canal sealers play a key role in creating a hermetic barrier between the canal wall and root filling material and in preventing pathogenic microorganisms and their by-products from infiltrating the canal space [2]. Reinfection of a root canal treated tooth is a leading cause of treatment failure and potentiates the need for laborious retreatment [3]. It is, therefore, important to evaluate the effectiveness of available and emerging sealers to both aid clinicians in making informed decisions about material selection and to contribute to the future research and development of endodontic sealers.

Nowadays, there are many sealers available of differing chemical compositions. AH Plus Jet^®^ Root Canal Sealer (AH Plus) is an epoxy-resin-based sealer currently in wide use, owing to its excellent physical and handling properties [2]. As a result, it is often used as a benchmark to compare other formulations against. It is, however, not without its drawbacks, as illustrated by its reported mutagenicity, cytotoxicity, minimal biomimetic properties, and hydrophobicity, which specifically reduces its compatibility within hydrophilic canals [4].

Recently introduced calcium silicate-based sealers (CSS) are, by contrast, highly biocompatible and bioactive [5]. For their inclusion of bioactive ceramic materials, they are also commonly referred to as ‘bioceramic sealers’. These injectable and pre-mixed hydrophilic sealers contain calcium silicates, calcium phosphate monobasic, calcium hydroxide, zirconium oxide, and a thickening agent [6,7]. Bioceramic-based sealers are hydrophilic and insoluble, allowing them to utilize the moisture present in the dentinal tubules to initiate and complete their setting reaction [7,8]. These sealers bind to dentine through a process known as tubular diffusion, which results in mechanical interlocking and the formation of a bond [9]. Currently available CSSs contain different formulations of calcium silicate and have desirable and well-researched physicochemical properties [10]. Examples of these include good bond strength to dentine as well as the ability to stimulate hard tissue growth as a result of the precipitation reaction of calcium silicate with available phosphate at the dentine-sealer interface [5]. Whilst these therapeutic effects are promising, more widespread adoption of CSSs is currently being limited by their questionable sealing ability, owing to their greater reported solubility.

It is evident that the performance of a sealer is dependent on both its chemical composition and physical properties [2]. These include its ability to penetrate into dentinal tubules as well as the strength of its bond to dentine [2]. Increased penetrability is thought to reduce microleakage by coating, thereby minimising untreated canal surfaces [3], while greater bond strength can improve the resistance of the sealer-core material complex to dislodgement under the dynamic and static forces exerted on teeth and their roots during normal function [11]. 

Thus, this study was to evaluate the properties of penetrability and bond strength for three different sealers. The sealers assessed in this study were AH Plus^®^ Bioceramic Sealer (AH Plus Bioceramic Sealer), a novel CSS introduced in 2021; EndoSequence^®^ BC Sealer (EndoSequence), a contemporary CSS; and AH Plus. While CSSs have already been extensively evaluated against other sealers, there is currently a lack of research comparing AH Plus Bioceramic Sealer to other commercially available sealers due to the recency of its introduction. The null hypotheses to be tested were that there is no difference in penetrability among the three sealers and that there is no difference in bond strength among the three sealers.

An additional aim of this study was to determine whether a correlation exists between the penetrability and bond strength of a sealer. It is thought that increased penetration of a sealer into intratubular dentine creates sealer tags, which enhances mechanical retention and in turn positively affects the sealer’s bond strength to dentine [12]. However, this appears to be based more on logical inference rather than on empirical evidence as few studies have directly evaluated this link [12]. Despite this, manufacturers often claim that the increased penetrability of a sealer corresponds to improved bond strength and a greater overall sealing ability [13]. Thus, an additional null hypothesis tested was that there is no correlation between the penetrability and bond strength of a sealer.

## 2. Materials and Methods

### 2.1. Sample Selection

This study was designed as a randomised controlled trial and was approved by the University of Queensland Human Ethics Committee (Low and negligible Risk category; ID: 2021/HE001879) for the use of human teeth. For teeth already planned for extraction, written informed consent was obtained on the day of extraction. An online statistical calculator (http://statulator.com/SampleSize/ss1M.html accessed on 1 August 2021) was used to calculate the sample size. Two-tailed 5% significance level (α = 0.05), 95% confidence interval, 90% statistical power (β = 0.10), 1:1 ratio of specimen allocation in the experimental groups, standard deviation (based on previous results) = 5, and medium estimated effect size (d = 0.60), which indicated the need to include a minimum of 20 specimens (slices) in each group. A total of 39 single rooted premolars was collected and stored in 0.01% thymol solution. The teeth were rinsed with water and decoronated using a NSK S-Max PICO high speed turbine handpiece (Nakanishi Inc., Kanuma, Japan). A bucco-lingually oriented pre-operative radiograph of each tooth was taken to produce an estimated working length, which was confirmed with a size #15 K-file (Dentsply Maillefer, Ballaigues, Switzerland) and an additional radiograph. The inclusion criteria used was: 3–4 mm bucco-lingual canal width; 1–2 mm mesio-distal width at the cemento-enamel junction level; closed apex; and root curvature up to 10°. The root lengths were standardized to 12.0 ± 0.5 mm and cut using an Isomet 1000 diamond disc (Buehler, Lake Bluff, IL, USA).

### 2.2. Root Canal Preparation and Obturation

The roots were prepared in a crown-down fashion with the TruNatomy™ rotary filing system (Dentsply Sirona, Ballaigues, Switzerland) in accordance with recommended manufacturer instructions. After each instrument change, the root canals were irrigated with 4% NaOCl. All canals underwent a final irrigation of 3 mL of 15% EDTA and 3 mL of 4% NaOCl before being dried with absorbent paper points (Dentsply Sirona, Ballaigues, Switzerland). 

The roots were randomly allocated to three treatment groups (*n* = 13), each to be obturated with a different sealer: either AH Plus Jet (Dentsply DeTrey, Konstanz, Germany) EndoSequence (Brasseler USA, Savannah, GA, USA) or AH Plus Bioceramic Sealer (Dentsply Sirona, Johnson City, TN, USA). From each group, three roots were obturated with their allocated sealers mixed with 0.1% rhodamine B dye (Sigma-Aldrich, St Louis, MO, USA) for confocal laser scanning microscopy (CLSM) analysis. Root canal obturation was performed using a single-cone technique, then stored in 100% relative humidity at 37 °C for 7 days to mimic in vivo conditions to allow for the complete setting of the materials.

### 2.3. Section Preparation

All obturated teeth were entombed in epoxy resin and allowed to cure completely. The specimens were transversely sectioned using a diamond disk (Isomet 1000, Buehler, Lake Bluff, IL, USA) at 350 RPM with a 450 g counterweight under copious water cooling. For each specimen, nine transverse slices of 0.9 mm thickness were obtained, with three slices allocated to each root third category (coronal, middle, and apical).

### 2.4. Confocal Laser Scanning Microscopy Analysis

Slices from the nine specimens prepared for imaging were scanned at 24 frames per second using a Nikon C2+ confocal microscope (Nikon, Tokyo, Japan) at ×10 magnification. Each sample was excited at 561 nm to detect the rhodamine B dye, with the detector range set to 568 nm. A large-field image with the same light intensity and offset was recorded for each slice at a size of 1024 × 1024 pixels. Each image was imported into Adobe Photoshop software (Adobe Systems, San Jose, CA, USA). In a new layer, the area of the root cross-section was traced and filled, and the total number of pixels was acquired in the histogram tool. The area of the canal space in pixels was obtained in the same way. By subtracting the area of the canal space from that of the root cross-section, the dentine area in pixels was calculated. Afterward, areas within the canal space that did not contain sealer (i.e., the GP cross-section and voids) were filled in red to match the colour of the sealer. Using the colour range tool, the colour red was selectively isolated, with the number of red pixels in the selection registered in the histogram tool. The sealer-impregnated dentin area in pixels was calculated by subtracting the canal space from the total red area. By dividing the number of red pixels from the total dentine area in pixels, the percentage of the dentine impregnated by the sealer was calculated. These percentages were used to measure the penetrability of the sealers examined.

### 2.5. Push-Out Test 

Slices from the thirty remaining specimens were used to test bond strength via push-out testing. Each slice was attached to a universal testing machine (Instron Model 3365, Instron, Canton, MA, USA). Various pluggers ranging in diameter from 0.3 mm, 0.6 mm, and 1.0 mm, selected based on the size of the root canal space for each slice tested, were used to apply a compressive force at a crosshead speed of 0.5 mm/min. 

To calculate bond strength in megapascals [14], the recorded load at failure in Newtons (N) was divided by the lateral area of root canal space in square millimetres (mm^2^). The lateral area of the root canal space was derived using the formula for a conical frustum, which is as follows:

$$SL=(R+r)\sqrt{h^{2}+(R-r{)}^{2}}$$

where *SL* is the lateral area of the root canal space (mm^2^), *R* is the mean radius of the coronal canal [5], *r* is the mean radius of the apical canal [5], and *h* is the thickness of the slice [5,15].

### 2.6. Failure Mode Analysis

Three specimens per group were randomly selected for a failure mode analysis. Slices were evaluated post push-out test and categorised based on the type of failure that occurred, according to previous studies with similar methodologies [15,16]. The specimens were examined under a stereomicroscope (Leica Microsystems, Heerbrugg, Switzerland) and categorised as either: adhesive failure (canal was sealer-free, indicating breakage at the sealer-dentine interface); cohesive failure (canal covered with sealer, indicating breakage within sealer); and mixed failure (canal had both sealer-covered and sealer-free regions).

### 2.7. Statistical Analysis

Statistical analysis was performed using Jamovi software version 2.2.5.0 for Windows (Jamovi, Sydney, Australia), with the significance level set at α = 0.05. Datasets were analysed using the Shapiro–Wilke test to assess normality. Two-way analysis of variance (ANOVA) was performed to analyse the effect of sealer type and root third on the penetrability and bond strength. Tukey’s Honest Significant Difference (HSD) was used as post hoc test. Statistically significant correlations between penetrability and bond strength were assessed using Pearson’s correlation.

## 3. Results

### 3.1. Penetrability

**Figure 1:**
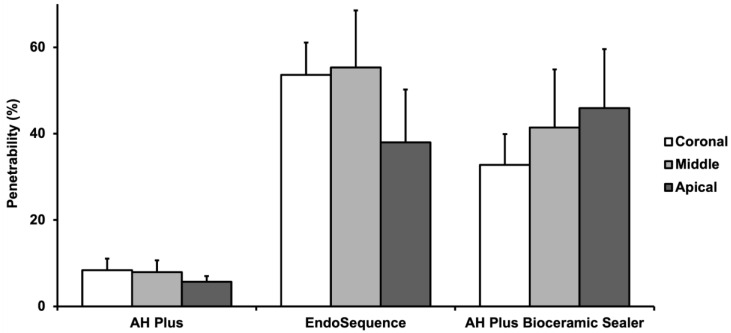
Comparison of mean penetrability (%) between sealers at different root canal thirds (coronal, middle, apical). There was no significant difference within the groups (One-way ANOVA-Tukey’s test post hoc, *p* > 0.05).

**Figure 2:**
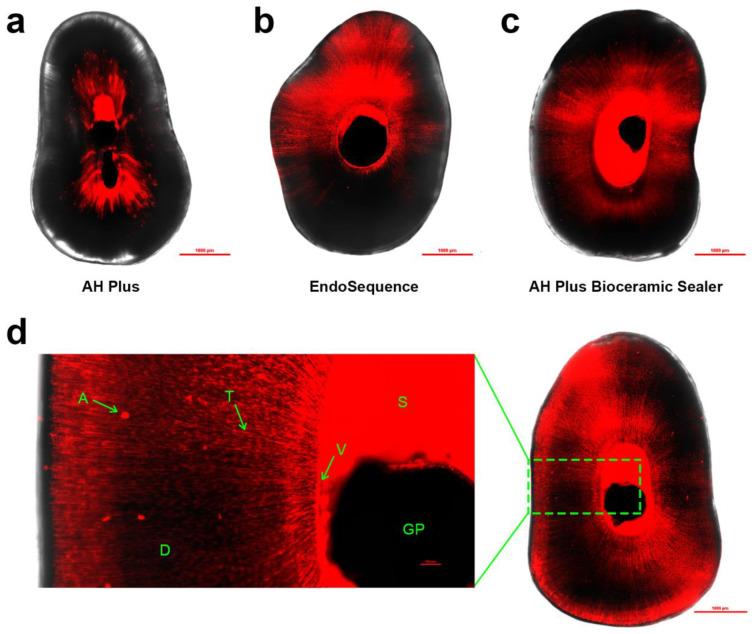
Representative CLSM images showing the penetrability of the three different root canal sealers: (**a**) AH Plus, (**b**) EndoSequence, and (**c**) AH Plus Bioceramic Sealer. (**d**) Higher magnification on a specimen obturated with EndoSequence showing additional features within the image: A: artefact (debris from cutting), T: sealer in dentine tubule, S: Sealer, V: void, D: dentine; GP: gutta-percha.

The Shapiro–Wilk test demonstrated a normal distribution of the data (*W* = 0.97, *p* = 0.06). Hence, parametric tests were used for the next steps. The mean penetrability (%) of each sealer stratified by root canal third is summarised in Figure 1 (Table S1. Supplementary). Two-way ANOVA revealed a significant difference among the sealer groups (*p* < 0.001). The effect of root canal third (*p* = 0.64) on penetrability was not significant, nor was the interaction effect (*p* = 0.30). Tukey HSD post hoc comparisons indicated that the mean penetrability of EndoSequence (50.34% ± 22.48%) and AH Plus Bioceramic Sealer (39.99% ± 23.36%) were statistically similar and that both were significantly greater than AH Plus (7.31% ± 4.65%). Representative CLSM images showing the penetrability of the three different root canal sealers are shown in Figure 2.

### 3.2. Bond Strength

**Figure 3:**
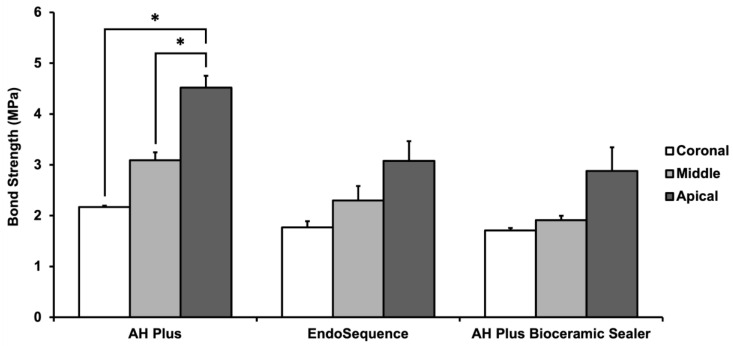
Comparison of mean bond strength [14] between sealers at different root canal thirds (coronal, middle, apical). * indicates a statistically significant difference (One-way ANOVA-Tukey’s test post hoc, *p* < 0.05).

The mean bond strength for each sealer stratified by root third is summarised in Figure 3 (Table S2—supplementary). Both sealer (*p* < 0.001) and root canal third (*p* < 0.001) had statistically significant effects on bond strength. The interaction effect was not significant (*p* = 0.29). The mean bond strengths of EndoSequence (2.38 ± 0.75 MPa) and AH Plus Bioceramic Sealer (2.16 ± 0.72 MPa) were statistically similar, and both were significantly lower than AH Plus (3.26 ± 1.06 MPa). Mean bond strength was consistently highest in the apical third and lowest in the coronal third when controlling for sealer type; however, significant differences were found within the AH Plus group only (Figure 3).

### 3.3. Failure Mode Analysis

The failure distribution in each root canal third for each of the sealers after the push-out test is displayed in Table 1. Cohesive failure was most prevalent in the AH Plus group (67%) and less prevalent in the EndoSequence (52%) and AH Plus Bioceramic Sealer groups (48%). For all sealers, more cohesive failures than adhesive failures occurred in the coronal and middle thirds, while more adhesive failures occurred in the apical third. Mixed failure occurred only once in the middle third with EndoSequence and once in the coronal third with AH Plus Bioceramic Sealer.

### 3.4. Correlation Analysis

The results of the correlation analysis using Pearson’s correlation, as seen in Table 2, found no significant correlation between penetrability and bond strength among root canal thirds for all three sealers compared (*p* > 0.05). There was also no significant correlation between penetrability and bond strength among the sealer groups (*r*(1) = −0.91, *p* = 0.269)

## 4. Discussion

This study assessed and compared the penetrability and bond strength of AH Plus, EndoSequence, and AH Plus Bioceramic Sealer. Additionally, the correlation between the penetrability of a sealer and its bond strength to dentine was evaluated.

To effectively function as an endodontic sealer, a material must possess adequate penetration into the dentine tubules, along with biocompatibility and sufficient mechanical properties [17]. However, even with the use of endodontic irrigants, such as NaOCl, chlorhexidine, calcium hydroxide, and EDTA, the sealer’s penetration into the dentine tubules is restricted [18,19,20]. Moreover, chemical disinfection and mechanical debridement of the root canal may damage the integrity of dentine tubules, leading to microleakage of bacteria, oral fluids, and other contaminants into the dentine [21]. Although the limited bond strength of the sealer–dentine interface causes marginal leakage, which may cause recurrent caries in endodontically treated teeth [22]. In this study, we used NaOCl and EDTA for disinfection and smear layer removal. Our findings indicated that EndoSequence and AH Plus Bioceramic Sealer both demonstrated significantly higher penetrability than AH Plus; thus, the null hypothesis was rejected. This is important since the remnants of the sealer acted as a mechanical barrier between the intracanal disinfectants and the microorganisms in areas that were difficult to reach, such as the dentinal tubules and ramifications [23]. From another perspective for non-surgical root canal retreatments, their apical retrieval may assist the clinician to attain apical patency. A number of studies have investigated the removal of bioceramic sealers for retreatment [24,25,26,27,28] and demonstrated a feasible reestablishment of patency and working length with an amount of remanent in the dentinal tubules [29]. 

The choice of investigating and presenting penetrability as a percentage of dye-impregnated dentine over the dentine area was similar to a study by Jardine et al. [30]. There are many previous studies that measure penetration by maximum depth of penetration in millimetres measured from the canal wall [3,31], while others used a limited number of depth measurements at regular intervals around the circumference of the canal to calculate an average [32,33]. Jardine et al. postulated that maximal penetration depths may not be representative of overall penetrability, as penetration is generally not homogenous along the perimeter of the central canal [30]. Consequently, to provide a more representative measure of overall penetrability, the percentage of sealer-impregnated dentine was the chosen means of measurement in this study. Similar to other similar studies, the use of rhodamine B dye allowed effective visualisation of sealer penetration due to the high contrast present when imaged with the software Nikon NIS [30,32]. The dye mix was maintained at 0.1% rhodamine B, in accordance with Hachem et al.’s observations that dye concentrations < 0.2% would not affect the physical properties of the sealers [32]. Despite this, in this study it was apparent that the dye did noticeably decrease the sealer viscosity when combined with EndoSequence and AH Plus Bioceramic Sealer. Referencing Figure 3, the nature of sealer penetration appears markedly different in the images, representative of the EndoSequence and AH Plus Bioceramic Sealers compared to that of AH Plus, with sealer appearing to infiltrate large areas of the cross section and occasionally pooling along the roots’ surfaces. The change in viscosity of the two CSSs sealers likely resulted in an overestimation of their penetrability compared to AH Plus.

Nonetheless, the finding that the penetrability of EndoSequence was greater than AH Plus was in support of similar studies reporting the former’s deeper penetration depths compared to the latter [32,34]. Al Haddad et al. suggested this observation was the result of the alkaline by-products of setting reaction of CSSs denaturing collagen fibres in dentine and enhancing its penetration into dentinal tubules [35].

In contrast, Eskander et al. reported deeper penetration scores in their study with AH Plus compared to EndoSequence [33]. This difference may be due to their use of lateral condensation and accessory GP cones, which produced condensing forces within the canals that compressed sealers towards the peripheral walls; thus, aiding AH Plus in adapting and penetrating dentinal tubules.

In terms of bond strength, overall, our results demonstrated that AH Plus had superior push-out strength compared to EndoSequence and AH Plus Bioceramic sealers. Although the CSSs had other favourable attributes that contributed to its sealing ability due to deeper penetration and low polymerization shrinkage, perhaps the most significant quality of AH Plus would be the ability of its epoxide rings to bind with amino groups of dentinal collagens [36,37]. 

The higher bond strength of AH Plus compared to other CSSs has been presented in various previous studies [15,38,39]. Cohesion amongst epoxy-resin-based sealer molecules and their ability to form covalent bonds between their epoxide rings and the exposed amino groups found in the collagen in dentine have been used to explain the excellent adhesion properties of AH Plus [40,41].

In contrast, Garlapati et al. found that CSSs had stronger bond strength values when compared to AH Plus [42]. Whilst some studies have indicated that CSSs possessed slightly lower flow rates than resin-based sealers [33], they were reported to have greater chemical and physical dentine adhesion compared to AH Plus [5]. Once mixed, CSSs reacted with available tissue fluids in a hydraulic setting reaction, hardening and expanding within the canal system to form a physical seal [5]. Simultaneously, the precipitation of hydroxyapatite-like crystals at the sealer-dentinal interface created a chemical bond, further improving bond strength [5].

Cohesive failure was the predominant pattern in the AH Plus group. Similar results were observed by Tedesco et al. [15], which may be associated with the strong bond strength exhibited by AH Plus in this current report and other previous studies [15,38,39]. Both CSSs within this study had more overall adhesive failures than AH Plus, which aligned with results found by Tedesco et al. and Donnermeyer et al. [15,43].

Similar to existing literature, no significant correlation was found between penetrability and bond strength [15,38]. Thus, the third null hypothesis was accepted. Tedesco et al. suggested that the relationship between these two physical properties was not linear but complex and hinged on multiple additional factors [15].

A major limitation of this study was its small sample size in comparison to previous studies with similar methodologies [15,30,32]. This, in turn, reduced the power and generalisability of the results obtained, especially for the correlation analysis. The other limitation was using rhodamine B. Although there are many studies that used rhodamine B for penetrability studies of sealers, due to water avidity by rhodamine B, it seems fluorophore stains (Flu-3) instead of rhodamine B could be more accurate [44,45]. However, since rhodamine B stains are commonly used, we also used the same protocol in this study, as it facilitated our comparisons. In addition, this study was limited to examining single-rooted premolars to control for confounding factors such as anatomical discrepancies. Future studies could incorporate multi-rooted and molar teeth to assess the versatility and effectiveness of sealers in varying canal systems, particularly multi-rooted systems.

## 5. Conclusions

The penetrability of EndoSequence and AH Plus Bioceramic Sealer was found to be higher than AH Plus, and the bond strength of AH Plus was higher than that of EndoSequence and AH Plus Bioceramic Sealer. Within the limitations of this study, our findings indicate that both bioceramic-based root canal sealers performed better than the epoxy-resin-based sealer in terms of penetrability. No correlation was found between penetrability and bond strength. Further research on the biomechanical properties, particularly setting and bioactivity, will be helpful to make a better comparison. 

## Figures and Tables

**Table 1:** Failure distribution (n) in each root canal third for each sealer after push-out test.

Sealer	Root Canal Third	Adhesive Failure	Cohesive Failure	Mixed Failure
AH Plus	Coronal	1 (11%)	8 (89%)	0 (0%)
	Middle	2 (22%)	7 (78%)	0 (0%)
	Apical	6 (67%)	3 (33%)	0 (0%)
EndoSequence	Coronal	3 (33%)	6 (67%)	0 (0%)
	Middle	3 (33%)	5 (56%)	1 (11%)
	Apical	6 (67%)	3 (33%)	0 (0%)
AH Plus Bioceramic Sealer	Coronal	4 (44%)	4 (44%)	1 (11%)
	Middle	4 (44%)	5 (56%)	0 (0%)
	Apical	5 (56%)	4 (44%)	0 (0%)

**Table 2:** Pearson’s correlation coefficient and corresponding *p*-value between bond strength and penetrability among root canal thirds for each sealer.

Sealers	Correlation Coefficient	*p*-Value
AH Plus	−0.97	0.162
EndoSequence	−0.92	0.254
AH Plus Bioceramic Sealer	0.86	0.343

## Data Availability

Not applicable.

## Supplementary Materials

The following supporting information can be downloaded at: https://www.mdpi.com/article/10.3390/ma16093309/s1, Table S1: Means and standard deviations of penetrability (%) as a function of sealer and root third and their main effects. Table S2: Means and standard deviations of bond strength [14] as a function of sealer and root third and their main effects.
