# Supplementary material for: Dentinal Tubule Penetrability and Bond Strength of Two Novel Calcium Silicate-Based Root Canal Sealers
Source: Materials (Basel). 2023 Apr 23;16(9):3309. doi: 10.3390/ma16093309 (PMC10179649; doi:10.3390/ma16093309)
Supplement: Supplementary file 1 [file materials-16-03309-s001.zip › materials-2276963-supplementary.pdf]

Supplementary Tables

Table S1—supplementary. Means and standard deviations of penetrability (%) as a function of sealer and root third and their main effects

	Root Third – Mean (SD)			
Sealer	Coronal	Middle	Apical	Marginal Means
AH Plus	8.35 (5.42)	7.94 (5.47)	5.70 (2.65)	7.31 (4.65) a,b
EndoSequence	53.60 (15.00)	53.33 (26.41)	37.95 (24.52)	50.34 (22.48) a
AH Plus Bioceramic Sealer	32.74 (14.26)	41.34 (26.85)	45.89 (27.37)	39.99 (23.36) b
Marginal Means	33.42 (22.05)	34.87 (29.26)	29.84 (27.32)	

*Same lowercase letters denote significant differences between root canal sealers (p <0.05 by post-hoc Tukey's test (n=10)).

Table S2—supplementary. Means and standard deviations of bond strength [14] as a function of sealer and root third and their main effects

	Root Third – Mean (SD)			
Sealer	Coronal	Middle	Apical	Marginal Means
AH Plus	2.17 (0.05)	3.09 (0.31)	4.52 (0.47)	3.26 (1.06) a,b
EndoSequence	1.77 (0.24)	2.30 (0.57)	3.08 (0.77)	2.38 (0.75) a
AH Plus Bioceramic Sealer	1.71 (0.10)	1.91 (0.17)	2.88 (0.93)	2.16 (0.72) b
Marginal Means	1.88 (0.25) A,B	2.44 (0.62) B	3.49 (1.01) A	

*Same lowercase letters denote significant differences between root canal sealers (p <0.05 by post-hoc Tukey's test (n=10)).

*Same uppercase letters denote significant differences between root thirds (p <0.05 by post-hoc Tukey tests (n=30)).
